# The effect of congenital blindness on resting-state functional connectivity revisited

**DOI:** 10.1038/s41598-021-91976-9

**Published:** 2021-06-14

**Authors:** Maria J. S. Guerreiro, Madita Linke, Sunitha Lingareddy, Ramesh Kekunnaya, Brigitte Röder

**Affiliations:** 1 Biological Psychology and Neuropsychology, Institute for Psychology, University of Hamburg, Von-Melle-Park 11, 20146 Hamburg, Germany; 2 Biological Psychology, Department of Psychology, Carl Von Ossietzky University of Oldenburg, 26111 Oldenburg, Germany; 3 Department of Radiology, Lucid Medical Diagnostics, Banjara Hills, Hyderabad, Telengana 500082 India; 4 Child Sight Institute, Jasti V. Ramanamma Children’s Eye Care Center, Department of Pediatric Ophthalmology, Strabismus, and Neuro-Ophthalmology, L. V. Prasad Eye Institute, Kallam Anji Reddy Campus, Hyderabad, Telengana 500034 India

**Keywords:** Auditory system, Somatosensory system, Visual system

## Abstract

Lower resting-state functional connectivity (RSFC) between ‘visual’ and non-‘visual’ neural circuits has been reported as a hallmark of congenital blindness. In sighted individuals, RSFC between visual and non-visual brain regions has been shown to increase during rest with eyes closed relative to rest with eyes open. To determine the role of visual experience on the modulation of RSFC by resting state condition—as well as to evaluate the effect of resting state condition on group differences in RSFC—, we compared RSFC between visual and somatosensory/auditory regions in congenitally blind individuals (*n* = 9) and sighted participants (*n* = 9) during eyes open and eyes closed conditions. In the sighted group, we replicated the increase of RSFC between visual and non-visual areas during rest with eyes closed relative to rest with eyes open. This was not the case in the congenitally blind group, resulting in a lower RSFC between ‘visual’ and non-‘visual’ circuits relative to sighted controls only in the eyes closed condition. These results indicate that visual experience is necessary for the modulation of RSFC by resting state condition and highlight the importance of considering whether sighted controls should be tested with eyes open or closed in studies of functional brain reorganization as a consequence of blindness.

## Introduction

Resting-state functional connectivity (RSFC) has increasingly been used to investigate functional brain organization in humans^1–3^. Initially motivated by the observation that spontaneous fluctuations of the blood-oxygen-level-dependent (BOLD) signal are not randomly organized, but appear to be temporally correlated between spatially distributed regions with similar functionality^4,5^, these studies have identified a number of primary resting-state networks (e.g., medial visual, somatosensory-motor, auditory)^6–8^, which closely resemble patterns of co-activation observed during task-based functional magnetic resonance imaging (fMRI) studies^8,9^.

Although early studies have suggested that patterns of RSFC are largely consistent across resting state conditions (i.e., whether participants rested with their eyes open or closed)^10,11^, subsequent studies have indicated that RSFC can, in fact, be modulated by resting state condition^2,12–26^. In particular, recent studies seem to converge on the notion that RSFC between visual and non-visual (i.e., somatosensory-motor, auditory) neural systems is increased when participants rest with their eyes closed, an effect that appears to be reduced or even reversed when participants rest with their eyes open^16,18,21,23,25,26^. In contrast, some studies have suggested that RSFC within the visual system is increased during rest with eyes open relative to rest with eyes closed^18,21,25^. Furthermore, studies using graph theory approaches to investigate the organization of whole-brain functional networks across resting states have provided evidence for higher global efficiency^21^, as well as lower average network connection distance^17^, during rest with eyes closed relative to rest with eyes open. In contrast, local efficiency and cliquishness were both found to be increased during rest with eyes open relative to rest with eyes closed^21^. Taken together, these results have been interpreted as indicating that eye closure acts as a toggle between a more specialized (or exteroceptive) mode of information processing during rest with eyes open and a more integrated (or interoceptive) mode of information processing during rest with eyes closed^21,26^.

The effect of resting state condition on RSFC (and derived measures of functional network topology) is consistent other known effects of resting state condition on brain organization. For example, resting-state brain activity (as measured by e.g. the amplitude of low-frequency fluctuations) has been shown to be higher in somatosensory and auditory regions—but lower in many visual regions—during rest with eyes closed relative to rest with eyes open^15,18,24,27–31^. Furthermore, block-design fMRI studies have provided evidence for increased activation in visual, somatosensory, and auditory cortical regions—but decreased activation in oculomotor and attentional regions—during blocks of eyes closed relative to blocks of eyes open^32–34^. Finally, studies using electroencephalography (EEG) have provided evidence for enhanced posterior alpha activity during rest with eyes closed relative to rest with eyes open^35,36^.

Importantly, the role of visual experience on the modulation of RSFC across resting state conditions remains hitherto unknown. A previous resting-state fMRI study in the normally sighted population has suggested that the effects of resting state condition (i.e., eyes open *vs.* eyes closed) on different parameters of brain activity are independent of exogenous visual input^17^, as they were shown to occur regardless of visual input (i.e., lights on *vs.* lights off). Accordingly, a recent study has found no evidence for an effect of congenital blindness on the modulation of resting-state brain activity across resting state conditions, inasmuch as none of the regions where an effect of resting state condition was found exhibited a significant Condition × Group interaction^37^. Two other lines of evidence do, however, suggest that visual experience may be necessary for patterns of RSFC to be modulated by resting state condition. First, EEG studies have revealed that the enhanced posterior alpha activity which is typically observed in sighted individuals during rest with eyes closed (relative to rest with eyes open)^35,36^ is reduced in blind individuals^35,38,39^. Second, a block-design fMRI study has shown that the increased activation in visual, somatosensory, and auditory cortical regions that is observed in sighted individuals during blocks of eyes closed (relative to blocks of eyes open)^32–34^ is largely attenuated in congenitally blind individuals^40^.

Interestingly, a growing number of studies examining the effect of congenital or early-onset blindness on RSFC have provided converging evidence for lower RSFC between ‘visual’ and non-‘visual’ (i.e., somatosensory-motor, auditory) sensory cortical regions in blind individuals relative to sighted controls^41–52^. Some of these studies have additionally provided evidence for lower inter-hemispheric RSFC within ‘visual’ regions, particularly within extrastriate cortex^41,43,44,46,47,51,53–55^. Because these studies have (understandably) strived to compare blind individuals and sighted controls under perceptually equivalent conditions (i.e., eyes closed)—and because, as reviewed above, RSFC between visual and non-visual sensory systems has been shown to be increased in sighted individuals during rest with eyes closed^16,18,21,25,26^—, this raises the intriguing possibility that previously reported group differences^41–52^ may have been due to a state-dependent enhancement of RSFC in sighted individuals during rest with eyes closed, rather than due to a genuine reduction of RSFC in congenitally blind individuals. Indeed, to truly demonstrate that RSFC between ‘visual’ and non-‘visual’ sensory cortices is reduced in congenital blindness requires demonstrating that such effects are independent of whether RSFC is compared between groups during rest with eyes closed or during rest with eyes open.

The goal of the present study was, therefore, twofold. First, we aimed to examine the role of visual experience on the modulation of RSFC by resting state condition (i.e., eyes open *vs.* eyes closed). To this end, we evaluated whether the effect of resting state condition on RSFC between ‘visual’ and non-‘visual’ sensory cortices differs between a group of congenitally blind individuals (*n* = 9) and a group of age- and gender-matched sighted participants (*n* = 9). In doing so, this study critically allowed us to additionally assess whether group differences in RSFC between congenitally blind individuals and sighted controls depend on resting state condition (second goal). Importantly—because different acquisition parameters and analytical strategies have been proposed to affect the results and interpretation of RSFC studies^3^—, we started by probing the validity of our methodological approach (e.g., parcellation scheme) in an initial study conducted in a larger sample of sighted participants (*n* = 28), which aimed to replicate the effect of resting state condition on RSFC between visual and non-visual sensory cortical regions in the normally sighted population^16,18,21,25,26^.

We hypothesized that sighted controls, but not congenitally blind individuals, would show a significant increase in RSFC between visual and non-visual sensory cortices during rest with eyes closed relative to rest with eyes open^16,18,21,25,26^. Furthermore, we hypothesized that group differences in RSFC between congenitally blind individuals and sighted participants depend on resting state condition, such that they are observed during rest with eyes closed^41–52^, but not—or to a lesser extent—during rest with eyes open.

## Results

### Study 1

In this study, we investigated the effect of resting state condition (i.e., eyes open *vs.* eyes closed) on RSFC between visual and somatosensory cortices, as well as between visual and auditory regions, in a group of healthy individuals with normal or corrected-to-normal vision (*n* = 28).

**Figure 1:**
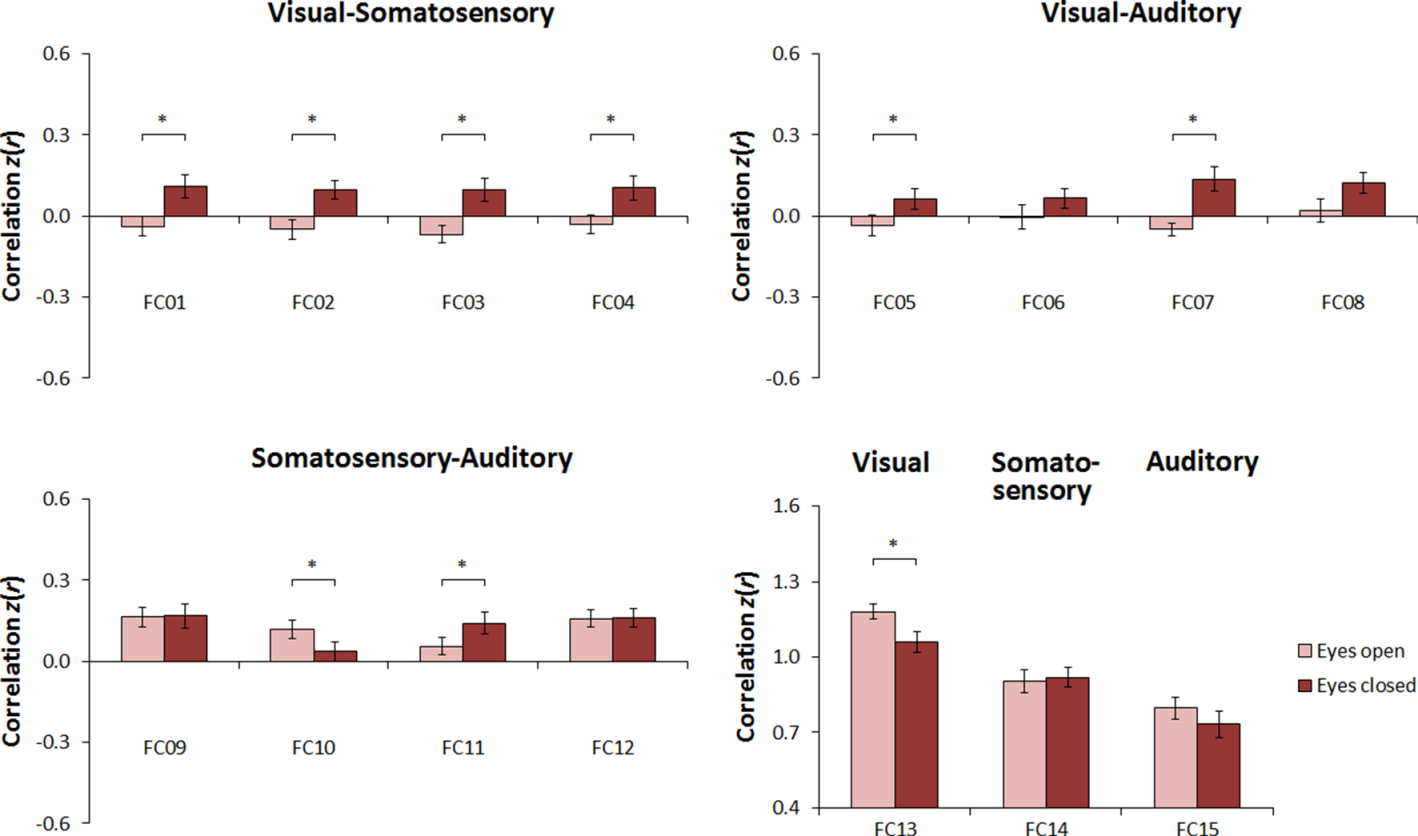
Effects of resting state condition in sighted individuals in Study 1. Shown is the average functional connectivity with the standard error of the mean in the eyes open condition (light red bars) and in the eyes closed condition (dark red bars) for each instance of functional connectivity examined (for details, see Table 1).

As expected^16,18,21,25,26^, paired-sample *t*-tests revealed a significant effect of resting state condition in most instances of RSFC examined (Fig. 1; see also Table 1). The most predominant effect was that of an increase in RSFC during rest with eyes closed relative to rest with eyes open, particularly between visual and somatosensory regions, but also between visual and auditory regions. Post-hoc comparisons (i.e., one-sample *t*-test, separately by condition) revealed that RSFC was generally nonsignificant during rest with eyes open, but tended to become significantly positive during rest with eyes closed (Supplementary Table S1). In contrast to these effects, but again as expected^18,21,25^, RSFC between left and right visual regions increased during rest with eyes open relative to rest with eyes closed (Fig. 1; see also Table 1), being significantly positive in both of these conditions (Supplementary Table S1). There was no significant correlation between the strength of (any of) these effects and age (Table 1).

**Table 1:** Summary of statistical analyses (*t* and *r* values) in Study 1.

RSFC instance	Region 1	Region 2	*n*^a^	Condition^b^	Age^c^
FC01	VIS_LH	SOM_LH	28	3.67*	0.10
FC02	VIS_LH	SOM_RH	28	4.22*	0.08
FC03	VIS_RH	SOM_LH	28	3.86*	0.22
FC04	VIS_RH	SOM_RH	27	3.10*	0.23
FC05	VIS_LH	AUD_LH	28	2.29*	− 0.14
FC06	VIS_LH	AUD_RH	28	1.53	− 0.36
FC07	VIS_RH	AUD_LH	23	4.52*	0.09
FC08	VIS_RH	AUD_RH	28	2. 19	− 0.25
FC09	SOM_LH	AUD_LH	27	0.14	0.04
FC10	SOM_LH	AUD_RH	28	− 2.46*	− 0.08
FC11	SOM_RH	AUD_LH	28	2.49*	0.11
FC12	SOM_RH	AUD_RH	28	0.09	0.07
FC13	VIS_LH	VIS_RH	25	− 2.54*	− 0.31
FC14	SOM_LH	SOM_RH	28	0.32	0.13
FC15	AUD_LH	AUD_RH	28	− 1.26	0.17

RSFC = resting-state functional connectivity; FC = functional connectivity; VIS = visual region; LH = left hemisphere; SOM = somatosensory region; RH = right hemisphere; AUD = auditory region.

^a^Sample size after outlier removal. ^b^Positive *t* values indicate that the mean was higher in the eyes closed condition than in the eyes open condition, whereas negative *t* values indicate that the mean was lower in the eyes closed condition than in the eyes open condition. ^c^Positive *r* values indicate that the mean difference between the eyes closed condition and the eyes open condition was higher with increasing age, whereas negative *r* values indicate that the mean difference between the eyes closed condition and the eyes open condition was lower with increasing age. *p < .050 (FDR corrected).

The results of Study 1 replicated those of recent reports showing that RSFC between visual and somatosensory cortical regions, as well as between visual and auditory cortical regions, is increased in both sighted adolescents^25^ and sighted adults^16,18,21,26^. Importantly, we replicated these effects despite substantial differences in the parcellation scheme used here and those used in previous reports^16,21,25^, indicating that our approach is adequate for investigating functional connectivity between sensory systems defined at the level of primary resting-state networks. Accordingly, a similar—though not as strong—pattern of results emerged when using a different parcellation scheme (see Supplementary Material), further corroborating the adequacy of our approach for investigating the effect of resting state condition on RSFC between sensory cortices.

### Study 2

In this study, we investigated the role of visual experience on the modulation of RSFC across resting state conditions (i.e., eyes open *vs.* eyes closed), by comparing a group of congenitally blind individuals (*n* = 9) with a group of age- and gender-matched sighted participants (*n* = 9). Most important, we additionally examined whether group differences in RSFC between congenitally blind individuals and sighted controls depend on resting state condition (i.e., eyes closed or eyes open).

A Condition (2 levels: eyes open, eyes closed) × Group (2 levels: congenitally blind, sighted controls) repeated measures ANOVA revealed a significant main effect of group on RSFC between left and right visual regions (Table 2), indicating an overall lower inter-hemispheric RSFC within the visual cortex in congenitally blind participants (*M* = 0.76, *SE* = 0.05) than in sighted individuals (*M* = 1.04, *SE* = 0.05). Post-hoc comparisons (i.e., one-sample *t*-tests, collapsed across resting state conditions) revealed that inter-hemispheric RSFC within the visual cortex was significantly positive both in the sighted control group, *t*(8) = 20.64, *p* < 0.001, and in the congenitally blind group, *t*(8) = 13.33, *p* < 0.001. The main effect of group on RSFC between the right visual cortex and the left somatosensory cortex was likewise significant (Table 2), suggesting an overall lower RSFC in congenitally blind individuals (*M* = − 0.18, *SE* = 0.05) relative to sighted controls (*M* = 0.10, *SE* = 0.06); however, this effect was qualified by a significant Condition × Group interaction (see below). No other main effects of group, as well as no main effects of condition, reached significance.

**Table 2:** Summary of statistical analyses (*F* values) in Study 2.

RSFC instance	Region 1	Region 2	*n*^*a*^		Group	Condition	Condition × Group
			SC	CB			
FC01	VIS_LH	SOM_LH	9	9	2.54	0.04	7.98*
FC02	VIS_LH	SOM_RH	9	9	0.62	0.01	8.04*
FC03	VIS_RH	SOM_LH	6	9	10.88*	0.01	12.19*
FC04	VIS_RH	SOM_RH	9	9	1.85	0.01	4.25
FC05	VIS_LH	AUD_LH	9	9	0.02	0.01	6.37
FC06	VIS_LH	AUD_RH	9	9	0.08	3.66	2.02
FC07	VIS_RH	AUD_LH	9	9	1.20	0.37	8.61*
FC08	VIS_RH	AUD_RH	9	8	5.22	0.31	2.92
FC09	SOM_LH	AUD_LH	9	9	1.20	0.02	0.07
FC10	SOM_LH	AUD_RH	8	9	1.24	0.01	1.18
FC11	SOM_RH	AUD_LH	9	8	0.99	4.08	0.09
FC12	SOM_RH	AUD_RH	9	9	0.92	0.22	0.02
FC13	VIS_LH	VIS_RH	9	9	14.42*	0.03	1.92
FC14	SOM_LH	SOM_RH	9	9	0.03	1.64	9.09*
FC15	AUD_LH	AUD_RH	7	9	0.01	0.52	5.47

RSFC = resting-state functional connectivity; CB = congenitally blind; SC = sighted controls; FC = functional connectivity; VIS = visual region; LH = left hemisphere; SOM = somatosensory region; RH = right hemisphere; AUD = auditory region.

^a^Sample size after outlier removal. *p < .050 (FDR corrected).

**Figure 2:**
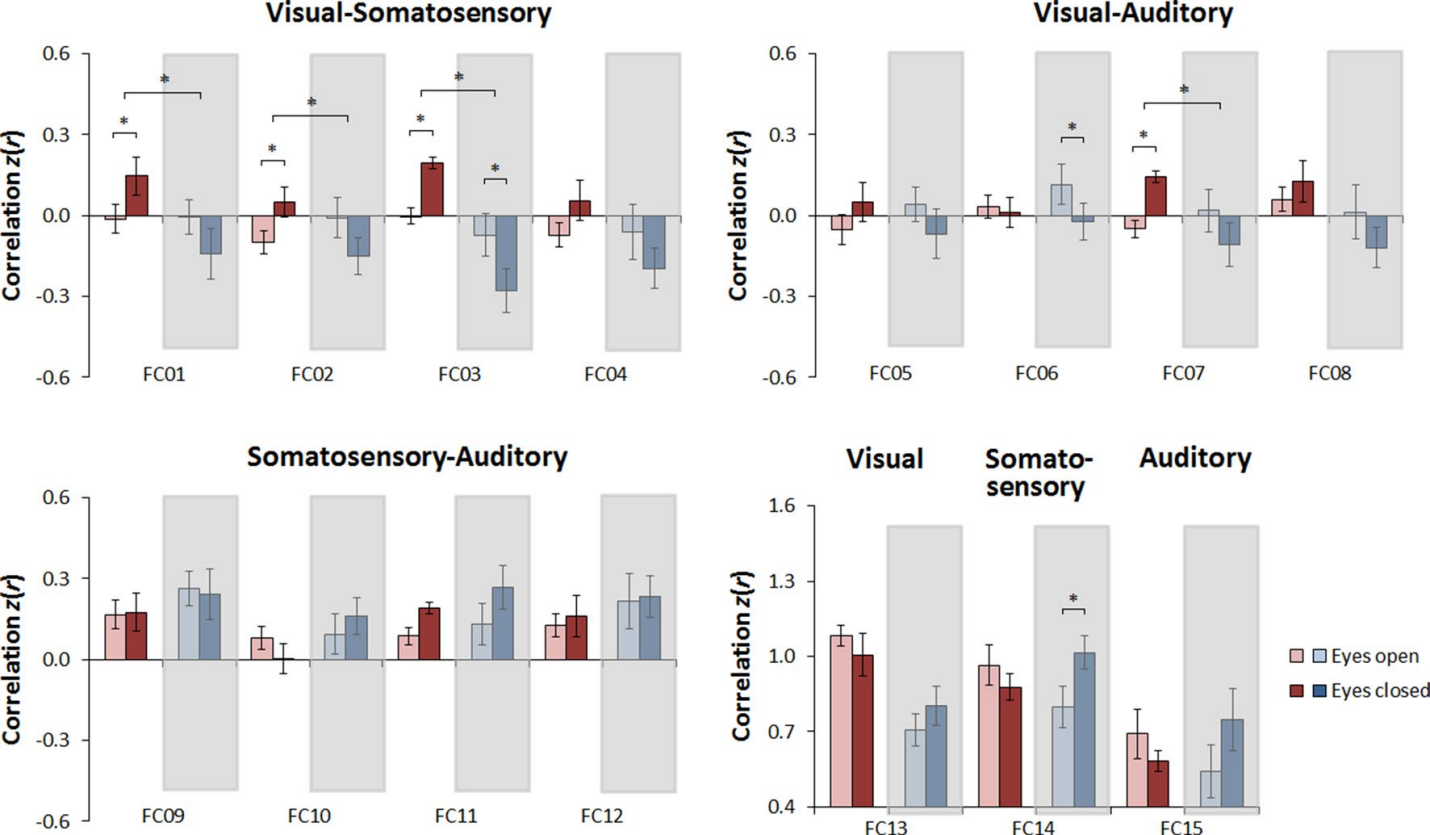
Effects of resting state condition in Study 2, separately by group (gray insets highlight the congenitally blind group). Shown is the average functional connectivity with the standard error of the mean in the eyes open condition (light red and light blue bars) and in the eyes closed condition (dark red and dark blue bars), plotted separately for normally sighted controls (red bars) and congenitally blind individuals (blue bars), for each instance of functional connectivity examined (for details, see Table 3). Note that this figure is equivalent to Fig. 3; here, the bars were organized to depict the effects of resting state condition separately by group.

Most important, a number of Condition × Group interactions were significant, particularly between the visual cortex and the somatosensory cortex, but also between the right visual cortex and the left auditory cortex (Table 2). Post-hoc comparisons (i.e., paired-sample *t*-tests, separately by group) revealed that most of these effects were driven by a significant increase in RSFC during rest with eyes closed relative to rest with eyes open in the sighted group, which was not observed in the congenitally blind group (Fig. 2; see also Table 3). Further post-hoc comparisons (i.e., one-sample *t*-tests, separately by group and condition) indicated that in the sighted group these effects were predominantly driven by nonsignificant RSFC during rest with eyes open that tended to become significantly positive during rest with eyes closed (cf. Supplementary Table S1), whereas in the congenitally blind group RSFC was mostly nonsignificant in both conditions (Supplementary Table S2).

**Table 3:** Summary of post-hoc, paired-sample *t*-tests in Study 2.

RSFC instance			Sighted controls		Congenitally blind	
			*n*^a^	Condition ^b^	*n*^a^	Condition^b^
FC01	VIS_LH	SOM_LH	9	3.73*	9	− 1.44
FC02	VIS_LH	SOM_RH	9	2.65*	9	− 1.65
FC03	VIS_RH	SOM_LH	6	4.61*	9	− 2.32*
FC04	VIS_RH	SOM_RH	9	1.68	9	− 1.32
FC05	VIS_LH	AUD_LH	9	1.99	9	− 1.65
FC06	VIS_LH	AUD_RH	9	− 0.30	9	− 2.86*
FC07	VIS_RH	AUD_LH	9	3.79*	9	− 1.32
FC08	VIS_RH	AUD_RH	9	0.76	8	− 1.82
FC09	SOM_LH	AUD_LH	9	0.12	9	− 0.25
FC10	SOM_LH	AUD_RH	8	− 1.11	9	0.61
FC11	SOM_RH	AUD_LH	9	1.35	8	1.48
FC12	SOM_RH	AUD_RH	9	0.68	9	0.18
FC13	VIS_LH	VIS_RH	9	− 0.84	9	1.13
FC14	SOM_LH	SOM_RH	9	− 1.25	9	2.99*
FC15	AUD_LH	AUD_RH	7	− 1.20	9	2.16

RSFC = resting-state functional connectivity; FC = functional connectivity; VIS = visual region; LH = left hemisphere; SOM = somatosensory region; RH = right hemisphere; AUD = auditory region.

^a^Sample size after outlier removal. ^b^Positive *t* values indicate that the mean was higher in the eyes closed condition than in the eyes open condition, whereas negative *t* values indicate that the mean was lower in the eyes closed condition than in the eyes open condition. *p < .050.

**Figure 3:**
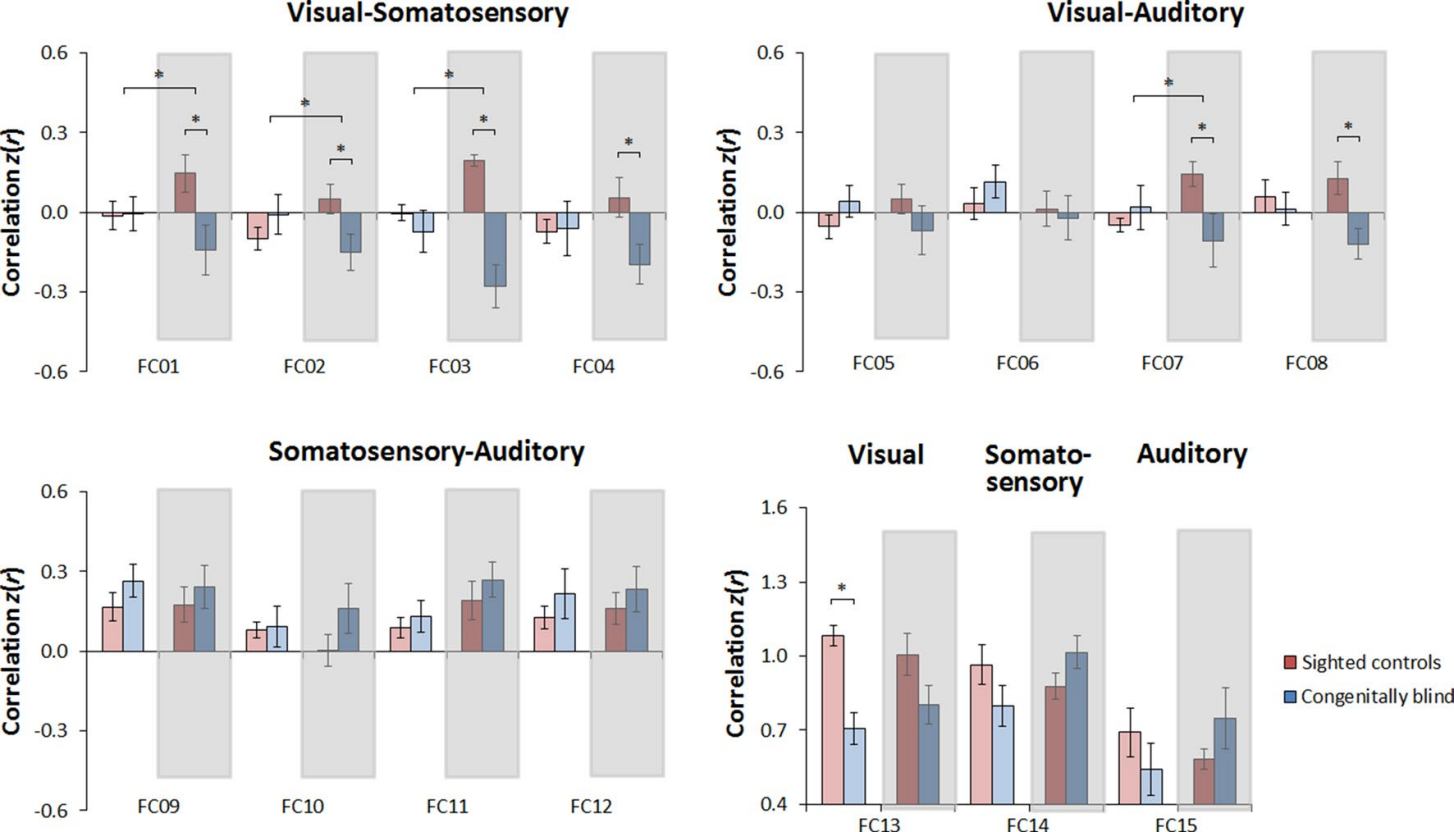
Effects of group in Study 2, plotted separately by resting state condition (gray insets highlight the eyes closed condition). Shown is the average functional connectivity with the standard error of the mean in the sighted group (red bars) and in the congenitally blind group (blue bars), plotted separately for the eyes open condition (light red and light blue bars) and the eyes closed condition (dark red and dark blue bars), for each instance of functional connectivity examined (for details, see Table 4). Note that this figure is equivalent to Fig. 2; here, the bars were reorganized to depict the effects of group separately by resting state condition.

Crucially, further post-hoc comparisons (i.e., independent-sample *t*-tests, separately by resting state condition) indicated that differences in RSFC between groups were exclusively observed during rest with eyes closed, but not during rest with eyes open (Fig. 3; see also Table 4). In other words, we replicated the previously observed pattern of lower RSFC between ‘visual’ and non-‘visual’ sensory cortical regions in congenitally blind individuals relative to sighted controls^41–52^ during rest with eyes closed, whereas no such differences between groups were observed during rest with eyes open.

**Table 4:** Summary of post-hoc, independent-sample *t*-tests in Study 2.

RSFC instance	Region 1	Region 2	*n*^a^		Eyes open^b^	Eyes closed^b^
			SC	CB		
FC01	VIS_LH	SOM_LH	9	9	− 0.10	2.46*
FC02	VIS_LH	SOM_RH	9	9	− 1.06	2.29*
FC03	VIS_RH	SOM_LH	6	9	0.85	5.67*
FC04	VIS_RH	SOM_RH	9	9	− 0.11	2.34*
FC05	VIS_LH	AUD_LH	9	9	− 1.25	1.11
FC06	VIS_LH	AUD_RH	9	9	− 0.96	0.32
FC07	VIS_RH	AUD_LH	9	9	− 0.77	2.26*
FC08	VIS_RH	AUD_RH	9	8	0.53	2.90*
FC09	SOM_LH	AUD_LH	9	9	− 1.20	− 0.65
FC10	SOM_LH	AUD_RH	8	9	− 0.15	− 1.38
FC11	SOM_RH	AUD_LH	9	8	− 0.63	− 0.79
FC12	SOM_RH	AUD_RH	9	9	− 0.89	− 0.71
FC13	VIS_LH	VIS_RH	9	9	4.88*	1.76
FC14	SOM_LH	SOM_RH	9	9	1.45	− 1.64
FC15	AUD_LH	AUD_RH	7	9	1.02	− 1.29

RSFC = resting-state functional connectivity; CB = congenitally blind; SC = sighted controls; FC = functional connectivity; VIS = visual region; LH = left hemisphere; SOM = somatosensory region; RH = right hemisphere; AUD = auditory region.

^a^Sample size after outlier removal. ^b^Positive *t* values indicate that the mean was higher in the normally sighted group than in the congenitally blind group, whereas negative *t* values indicate that the mean was lower in the normally sighted group than in the congenitally blind group. *p < .050.

## Discussion

The first goal of the present study was to investigate the role of visual experience on the modulation of RSFC by resting state condition. In addition, the present study aimed at evaluating whether previously reported group differences in RSFC between congenitally blind individuals and sighted controls depend on whether RSFC is assessed during rest with eyes open or during rest with eyes closed.

First, we replicated the typical change in RSFC as a function of resting state condition in a group of sighted individuals: While interhemispheric RSFC within visual cortex was higher during rest with eyes open than during rest with eyes closed^18,21,25^, RSFC between visual and somatosensory cortices—as well as between visual and auditory regions—was higher during rest with eyes closed than during rest with eyes open^16,18,21,25,26^. In most cases, RSFC between visual and non-visual brain regions was nonsignificant during rest with eyes open, but became significantly positive during rest with eyes closed. These results are consistent with those of block-design fMRI studies, showing increased activation in visual, auditory, and somatosensory cortical regions during blocks of eyes closed (relative to blocks of eyes open) in sighted individuals^32–34^. These findings—together with an increase in topological network properties denoting functional integration (e.g., increased global efficiency^21^, lower average connection distance^17^)—have been interpreted as reflecting an integrated mode of (multisensory) information processing during rest with eyes closed^21^. Moreover, given that increased RSFC between visual and non-visual sensory systems has been shown to be related to posterior alpha activity in sighted participants^23^, these results are additionally in line with those of EEG studies showing enhanced alpha activity during rest with eyes closed (relative to rest with eyes open) in sighted individuals^35,36^. Finally, the present results concur with those of other resting-state fMRI studies examining the effect of resting state condition on resting-state brain activity (e.g., amplitude of low-frequency fluctuations), as they have have shown a particularly consistent effect of resting state condition not only at the level of visual cortex but additionally on somatosensory and auditory cortices^15,18,24,27–31^.

In contrast to sighted controls—but in line with our hypothesis—, the effect of resting state condition on RSFC between ‘visual’ and non-‘visual’ sensory cortices was predominantly nonsignificant in congenitally blind individuals. A previous report in sighted participants had suggested that the modulation of resting-state brain activity across resting state conditions (eyes open *vs.* eyes closed) is independent of visual input^17^, a finding that was recently corroborated in a study comparing the effect of resting state condition on resting-state brain activity between congenitally blind individuals and normally sighted controls^37^. In contrast to these studies—which have primarily focused on resting-state brain activity—, here we show that developmental visual experience is necessary for a significant increase in RSFC between visual and non-visual sensory systems to emerge in the eyes closed condition relative to the eyes open condition. The present findings are, however, consistent with the results of previous EEG and fMRI studies, demonstrating that the enhanced alpha activity and the increased activations in visual, auditory, and somatosensory cortical regions observed in sighted individuals during blocks of eyes closed relative to blocks of eyes open^32,35^ were substantially reduced or absent in blind individuals^35,40^. Importantly, here we extend these findings by showing that visual experience appears to be necessary for the development of what has been proposed to be a more integrated mode of information processing (characterized by enhanced activation of visual and non-visual cortices, as well as higher functional connectivity between visual and non-visual sensory systems) that is induced by eye closure in sighted individuals^21,32^.

Interestingly, our observation of group differences in the modulation of RSFC between visual and non-visual sensory systems across resting state conditions (i.e., eyes open *vs.* eyes closed) is in line with the results of a recent study demonstrating that visual experience is likewise critical for the modulation of functional connectivity between visual and auditory regions across rest and (auditory) task states^56^. Whereas sighted controls exhibited lower functional connectivity between visual and auditory cortical regions during auditory processing than during rest (both of which were conducted with eyes closed), the reverse pattern was found in congenitally blind individuals. Based on the present RSFC findings for the visual and somatosensory cortices, we would predict to find similar results for a somatosensory task condition vs. rest with eyes closed.

If the effect of resting state condition on RSFC differs—as we have shown here—between congenitally blind individuals and sighted controls, then the type of resting state condition used in a study may have non-trivial effects on whether group differences in RSFC are observed or not. In line with this, we have replicated previous findings of lower RSFC between ‘visual’ and non-‘visual’ sensory cortices in congenitally blind individuals relative to normally sighted controls^41–52^, but only when groups were compared during rest with eyes closed. These results are consistent with those of an early positron emission tomography (PET) study showing that glucose metabolism in the medial occipital cortex in individuals with congenital or early-onset blindness resembles that of sighted controls tested during rest with eyes open, rather than that of sighted controls tested during rest with eyes closed^57^.

In contrast to the above state-dependent effects, we observed that RSFC within the ‘visual’ system was overall lower (but still significant) in congenitally blind individuals than in sighted controls, and that this effect appeared to be independent of resting state condition as it was not qualified by a Condition × Group interaction. This result corroborates previous studies^41,43,46,47,51,53–55^ showing that the full maturation of interhemispheric RSFC within the visual cortex is dependent on visual experience. This finding is reminiscent of structural differences in the splenium—the major pathway connecting left and right regions of the occipital cortex—in congenital blindness. Indeed, a number of studies have provided evidence for reduced volume or surface area^58,59^, as well as reduced fractional anisotropy^60–62^, in this callosal region in individuals with congenital or early-onset blindness relative to sighted controls.

In the present study, we have focused on differences in RSFC between visual and non-visual sensory systems across resting state conditions because these are the instances of RSFC where *both* the effect of resting state condition *and* the effect of congenital blindness have been shown to be most reliable—therefore allowing us to investigate whether the effect of resting state condition on RSFC depends on visual experience (first goal) and whether group differences in RSFC depend on resting state condition (second goal). Given that a number of resting-state fMRI studies have observed an effect of resting state condition on patterns of RSFC involving other functional networks (e.g., default mode network^2,15,19,25,26^, salience or attentional networks^2,19–22,25,26^), future studies could investigate whether such effects—in functional networks beyond sensory cortices—are likewise dependent on visual experience, as well as the extent to which such differences (if any) may contribute to the present results. Furthermore, given that the organization of whole-brain functional networks has likewise been shown to differ across resting state conditions^21^, future studies could investigate the extent to which topological network properties differ between congenitally blind individuals and normally sighted controls, as well as the extent to which such differences may depend on resting state condition. Based on the present and previous^56^ findings, we predict that whole-brain functional network organization is more likely to differ between congenitally blind individuals and sighted controls during rest with eyes closed—when congenitally blind individuals may exhibit a more segregated functional network organization than sighted controls—, with a potential reversal of these effects during auditory and tactile processing.

Finally, in the present study we have interpreted group differences as stemming primarily from differences in developmental visual experience, as have most studies comparing brain organization between congenitally blind individuals and normally sighted controls. The observation of numerical differences between congenitally blind individuals with light perception and congenitally blind individuals without light perception on the modulation of RSFC between visual and non-visual sensory cortices across resting state conditions (Supplementary Fig. S3)—as well as on group differences in RSFC between visual and non-visual sensory cortices during rest with eyes closed (Supplementary Fig. S4)—, appear to be consistent with this interpretation; however, we note that these observations should be followed up in larger samples of congenitally blind humans. It could alternatively be argued that the group differences found in the present study may have resulted from differences between groups in other factors, in particular eye movements and/or motion during scanning. Although some blind individuals are known to show oculomotor abnormalities, others do not (e.g., those with severe microphthalmia). Thus, it is unlikely that the consistent group differences observed in the present study could have resulted from some blind participants with oculomotor abnormalities. Furthermore, although differences in the amount of motion during scanning have been acknowledged to play an important role on group differences in RSFC^63^, this possibility cannot account for the present pattern of results for a number of reasons. First, we found no significant differences in mean framewise displacement between groups, *F*(1,16) = 1.36, *p* = 0.261, resting state conditions, *F*(1,16) = 1.25, *p* = 0.279, or their interaction, *F*(1,16) = 0.24, *p* = 0.632. Second, we reduced the potential confounding effects of motion on the data by regressing out variance associated with the six motion parameters obtained during motion correction. Third, we employed a censoring approach to further mitigate the impact of residual motion-induced artifacts on the data^63^.

In summary, the results of the present study replicate those of previous studies in the normally sighted population^16,18,21,25,26^, by showing that RSFC between visual and non-visual (i.e., somatosensory, auditory) sensory brain regions is increased during rest with eyes closed relative to rest with eyes open. Importantly, however, the results of the present study extend this previous finding in two novel ways: First, we show that these effects seem to depend on visual experience, as they were observed in sighted participants but not in congenitally blind individuals; second, we demonstrate that group differences in RSFC between ‘visual’ and non-‘visual’ sensory cortices depend on resting state condition, as they were observed during rest with eyes closed but not during rest with eyes open. To the extent that the significantly positive RSFC between visual and non-visual sensory systems during rest with eyes closed observed here and in previous studies^16,18,21,25,26^ reveal a more integrated mode of (multisensory) information processing in normally sighted individuals^21^, our results suggest that visual experience is necessary for the emergence of such an integrated processing mode by eye closure in the resting state. Finally, our study underscores the critical role of whether sighted controls are tested with eyes open or with eyes closed in studies of functional brain reorganization as a consequence of blindness.

## Methods

### Participants

Participants in Study 1 were 28 individuals with normal or corrected-to-normal vision (aged 6–56 years, *M* = 21.6, *SD* = 11.8, 9 females), who had been recruited as controls in the context of a larger research project. Participants in Study 2 were 10 individuals with congenital blindness of peripheral (ocular) origin (aged 9–39 years, *M* = 19.6, *SD* = 7.8, 3 females) and a subgroup of 10 sighted individuals from Study 1 who were matched to the congenitally blind participants on the basis of age and gender (aged 10–41 years, *M* = 20.4, *SD* = 8.2, 3 females). Importantly, one congenitally blind participant (who had anophthalmia) was excluded from the analyses because resting-state data had only been acquired in the eyes closed condition. The final sample of Study 2 therefore comprised nine congenitally blind individuals (aged 9–39 years, *M* = 20.0, *SD* = 8.1, 3 females) and nine age- and gender-matched sighted participants (aged 10–41 years, *M* = 20.8, *SD* = 8.6, 3 females).

In the congenitally blind group, blindness was due to microphthalmia (*n* = 5), Leber’s congenital amaurosis (*n* = 2), and phthisis bulbi and corneal opacities (*n* = 2). In addition, visual acuity in the congenitally blind group ranged from no light perception (*n* = 2) to ability to perceive light (*n* = 3), fixing and following light (*n* = 1), ability to report the location of light (*n* = 1), and counting fingers close to face (*n* = 1) (for one participant, we were unable to retrieve this information).

All participants were recruited from the local area of Hyderabad and surrounding regions, and data acquisition was conducted in accordance with the principles outlined in the Declaration of Helsinki (2013). Prior to being included in the study, participants received information about the procedures and were screened to ensure that they had no conditions that would preclude their participation in an MRI examination (e.g., metal implants, pregnancy, claustrophobia). Written informed consent was obtained from all participants (or from their parents or legal guardians, in case of underage participants) before scanning.

The study was approved by the Ethics Commission of the German Psychological Society (BR 09_2013), the Local Ethics Committee of the Faculty of Psychology and Human Movement Science of the University of Hamburg (Röder 10/2015), and the Ethics Committee of the L. V. Prasad Eye Institute (LEC 11-086 and LEC 12-15-124).

### Experimental design

Each participant underwent two resting-state runs, each of which lasted 8.5 min. Before each run, participants were instructed to keep their eyes closed (i.e., eyes closed condition) or to keep their eyes open in low-level illumination (i.e., eyes open condition). In addition, participants were asked to try not to think about anything in particular, to move as little as possible, and to refrain from falling asleep.

The order of the two resting state conditions was set out to be counterbalanced across subjects; however, due to miscommunication, 18 sighted participants (rather than 14) were scanned with their eyes open first and 10 sighted participants (rather than 14) were scanned with their eyes closed first. Likewise, 7 congenitally blind participants (rather than 5) were scanned with their eyes open first and 3 congenitally blind participants (rather than 5) were tested with their eyes closed first.

Due to technical limitations (scanning was performed in a private radiology department)—but similarly to the vast majority of studies investigating the effect of resting state condition on resting-state brain activity and/or RSFC in the normally sighted population^2,12–22,24–31^—, we have not used an eye-tracker during scanning to ensure that participants complied with the instructions to keep their eyes open or closed throughout scanning. However, previous studies which have investigated the effect of eyes open and eyes closed conditions in sighted individuals or in congenitally blind individuals, and which did use an eye tracker, suggest that both participant groups are able to keep their eyes open or closed as instructed^23,40^. Accordingly none of our blind participants reported feeling weird about the instruction to keep their eyes open or closed.

### Data acquisition

Images were acquired on a 1.5 T GE Signal HDxt scanner (General Electric, Milwaukee, WI, USA), equipped with an 8-channel head coil, at a healthcare facility (Lucid Medical Diagnostics, Hyderabad, India).

In each functional run, 256 T2*-weighted images were obtained using a gradient-echo echo-planar imaging (EP/GR) sequence. The scanned volume included 32–38 axial slices to cover the entire brain (repetition time [TR] = 2000 ms; echo time [TE] = 30 ms; flip angle [FA] = 90°; field of view [FOV] = 220 × 220 mm; in-plane matrix size = 64 × 64; slice thickness = 3 mm; interslice gap = 4 mm; interleaved acquisition in ascending order).

In addition, anatomical T1-weighted images were obtained using a 3D-spoiled gradient recalled (3D-SPGR) sequence. The scanned volume included 168–196 axial slices to cover the entire brain (TR = 15 ms; TE = 7 ms; inversion time = 500 ms; FA = 15°; FOV = 220 × 220 mm; in-plane matrix size = 512 × 512; slice thickness = 1.6 mm; interslice gap = 0.8 mm).

### Data processing

Functional and anatomical data were processed using BrainVoyager 2.8.2 (BrainInnovation, Maastricht, the Netherlands). After excluding the first four volumes of each functional run (to account for T1 saturation effects), functional data underwent default processing, which included slice scan time correction (cubic spline interpolation), head motion correction (trilinear/sinc interpolation, using the first volume as reference), and removal of linear and non-linear drifts using a high-pass temporal filter (GLM-Fourier, 2 sines/cosines), but no spatial smoothing. The resulting, preprocessed functional datasets were then co-registered to the anatomical images and transformed into Talairach space (trilinear interpolation), resulting in an interpolated functional voxel size of 3 × 3 × 3 mm.

Based on the normalized (i.e., Talairach-transformed) 3D anatomical datasets, ventricle and while matter regions were defined for each participant. The average time course in these two regions, as well as the six parameters obtained by rigid body head motion correction (i.e., three translations and three rotations), were regressed out of the functional data to mitigate potential sources of spurious (i.e., non-neural-related) correlations (e.g., physiological noise, motion). Regression of these signals was performed simultaneously and the residual volumes were then retained for functional connectivity analysis.

**Figure 4:**
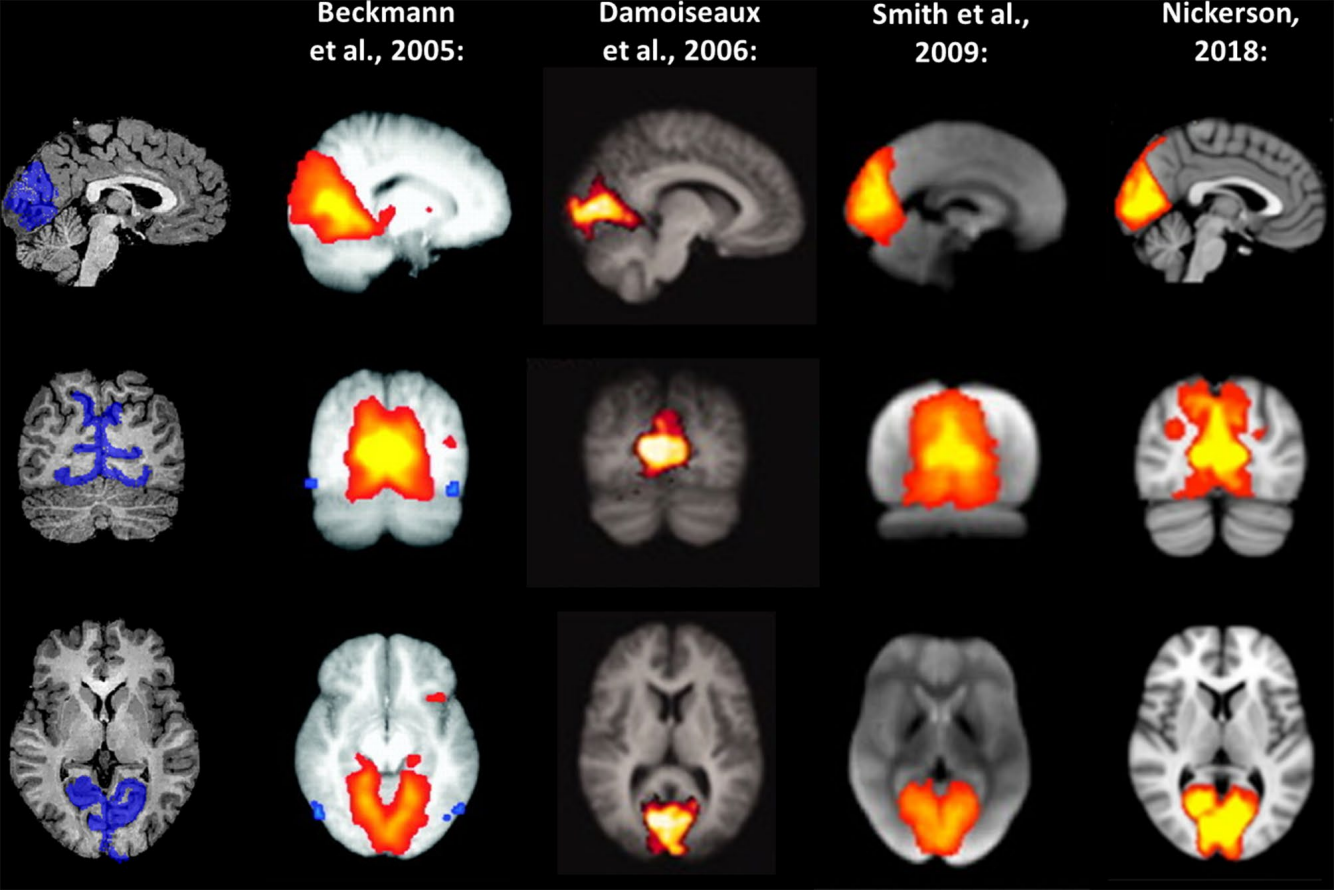
Left and right medial visual seeds (blue) used in the present study. For comparison, the medial visual system, as described in previous studies, is likewise depicted. Images modified from Beckmann et al.^6^, Damoiseaux et al.^7^, Smith et al.^8^, and Nickerson^9^, and generated using Microsoft® PowerPoint™.

**Figure 5:**
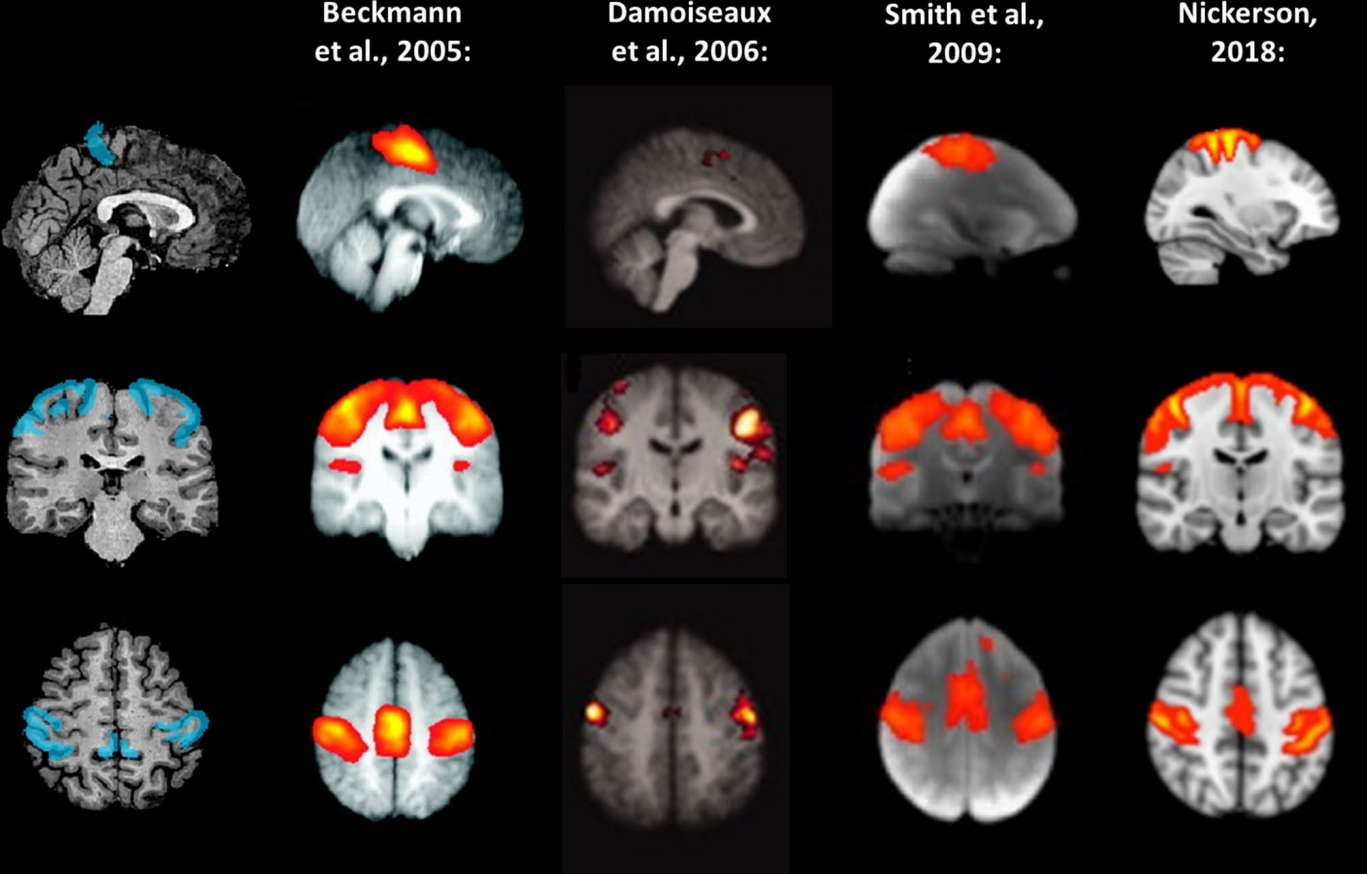
Left and right somatosensory seeds (cyan) used in the present study. For comparison, the sensory-motor system, as described in previous studies, is likewise depicted. Images modified from Beckmann et al.^6^, Damoiseaux et al.^7^, Smith et al.^8^, and Nickerson^9^, and generated using Microsoft® PowerPoint™.

**Figure 6:**
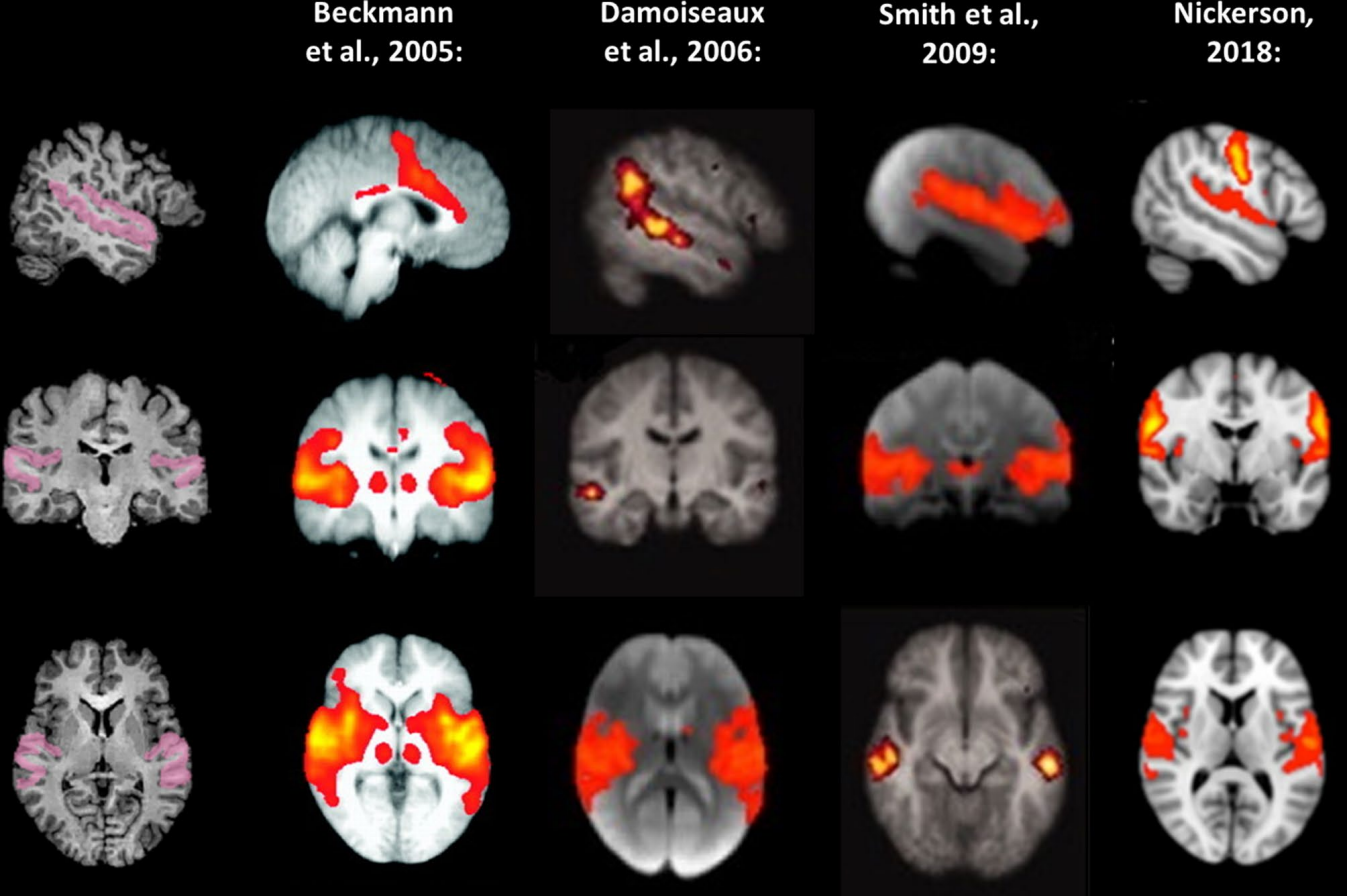
Left and right auditory seeds (pink) used in the present study. For comparison, the auditory system, as described in previous studies, is likewise depicted. Images modified from Beckmann et al.^6^, Damoiseaux et al.^7^, Smith et al.^8^, and Nickerson^9^, and generated using Microsoft® PowerPoint™.

Because our goal was to investigate RSFC between sensory brain systems—and in order to keep data dimensionality low (given the limited sample sizes inherent to studies involving special populations)—, we defined sensory systems in terms gray matter regions corresponding to well-known primary resting-state networks^6–9^, which have been shown to be highly consistent across individual participants^7^. Specifically, we defined the following regions, separately by hemisphere: a *medial visual region*, comprising the calcarine sulcus, the lingual gyrus and the cuneus (Fig. 4); a *somatosensory region*, comprising the central sulcus, the postcentral gyrus, and the postcentral sulcus (Fig. 5); and an *auditory region*, comprising the superior temporal gyrus and the superior temporal sulcus (Fig. 6). These seed regions were created by anatomically labeling gyri and sulci in each participant’s brain hemisphere after aligning the respective cortical surface reconstruction to the atlas brain provided by BrainVoyager QX (in which gyri and sulci have been predefined) and transforming them back to volume space by expanding them (− 1 to + 3 mm) along the vertex normal of the white matter-gray matter boundary. As this procedure makes use of cortical curvature information, it allows for an accurate remapping of predefined cortical regions form the atlas brain provided by BrainVoyager QX to each individual’s brain.

### Statistical analysis

Functional connectivity strength was quantified by calculating Pearson’s correlations between the average time courses from each pair of seed regions, resulting in 15 (6 × 5/2) unique connections. A Fisher’s *r*-to-*z* transformation was additionally applied to improve normality of the correlation coefficients. Importantly, in order to mitigate the impact of residual motion-induced artifacts, we employed a censoring approach in which we withheld volumes with excessive relative displacement from the analyses^63^. Specifically, framewise displacement was calculated as the sum of the absolute values of the differences in translational and rotational realignment estimates (after converting rotational estimates from degrees to millimeters by estimating displacement on the surface of a sphere with a 50-mm radius) between consecutive volumes^64^. Subsequently, volumes exceeding a study-specific framewise displacement threshold of 0.35 mm were removed, provided that a minimum of 150 uncensored volumes (equivalent to 5 min of data) remained for analysis^2^. No participant had to be excluded due to this criterion. Importantly, in order to avoid that different participants and/or conditions would have different time-course lengths—as well as to reduce any systematic differences in the phase of the run from which volumes would be removed—, we censored the same amount of volumes (*n* = 52) across participants and conditions, corresponding to those volumes that exceeded our framewise displacement threshold (if any) and an additional number of (up to 52) randomly selected volumes.

Prior to statistical analyses, *z*(*r*) values three median absolute deviations away from the median in either resting state condition and in either group were considered outliers^65,66^ and removed from the analyses. Outlier removal was performed separately for each of the 15 instances of RSFC examined, so as to retain as many participants for analyses as possible (since outliers in one instance of RSFC may not be outliers in another instance). This led to a sample size of *n* = 23–28 sighted participants in Study 1 (for details, see Table 1), as well as to a sample size of *n* = 8–9 congenitally blind participants and *n* = 6–9 sighted participants in Study 2 (for details, see Table 2).

In Study 1, *z*(*r*) values were submitted to a paired-sample *t*-test to examine the effect of resting state condition (2 levels: eyes open, eyes closed) on the strength of RSFC between each pair of seed regions. In order to correct for multiple comparisons, we used the Benjamini–Hochberg false discovery rate (FDR) approach^67^ to find a threshold that would restrict the expected proportion of false-positive errors to lower than 5%. Post-hoc comparisons (i.e., one-sample *t*-tests against zero) were performed to determine the significance and direction of RSFC in each resting state condition. In addition, we performed Pearson’s correlations between the age of the participants and the difference in RSFC across resting state conditions.

In Study 2, *z*(*r*) values were submitted to a repeated measures ANOVA with participant group (2 levels: congenitally blind, sighted controls) as a between-group factor and resting state condition (2 levels: eyes open, eyes closed) as a within-group factor. As in Study 1, we corrected for multiple comparisons using the Benjamini–Hochberg FDR approach^67^ at a rate of *q* < 0.05. Post-hoc comparisons were performed to determine whether the effect of resting state condition on RSFC differed across groups (i.e., paired-sample *t*-tests, separately by group), as well as to examine whether group differences in RSFC differed across resting state conditions (i.e., independent-sample *t*-tests, separately by resting state condition). Post-hoc comparisons were additionally performed to evaluate the significance and direction of RSFC in each resting state condition and in each group (i.e., one-sample *t*-tests against zero).

## Supplementary Information

Supplementary Information.

## Data Availability

The aggregated data that support the findings of this study are available upon reasonable request to the corresponding author. The raw data are not publicly available owing to potentially identifying information that could compromise participant privacy, as well as due to the lack of explicit consent from the participants for this purpose.

## References

[1] Fox MD, Raichle ME (2007). Spontaneous fluctuations in brain activity observed with functional magnetic resonance imaging. Nat. Rev. Neurosci..

[2] Van Dijk KRA (2010). Intrinsic functional connectivity as a tool for human connectomics: Theory, properties, and optimization. J. Neurophysiol..

[3] Power JD, Schlaggar BL, Petersen SE (2014). Studying brain organization via spontaneous fMRI signal. Neuron.

[4] Biswal B, Yetkin FZ, Haughton VM, Hyde JS (1995). Functional connectivity in the motor cortex of resting human brain using echo-planar MRI. Magn. Reson. Med..

[5] Cordes D (2000). Mapping functionally related regions of brain with functional connectivity MR imaging. Am. J. Neuroradiol..

[6] Beckmann CF, DeLuca M, Devlin JT, Smith SM (2005). Investigations into resting-state connectivity using independent component analysis. Philos. Trans. R. Soc. B.

[7] Damoiseaux JS (2006). Consistent resting-state networks across healthy subjects. Proc. Natl. Acad. Sci. U. S. A..

[8] Smith SM (2009). Correspondence of the brain’s functional architecture during activation and rest. Proc. Natl. Acad. Sci. U. S. A..

[9] Nickerson LD (2018). Replication of resting state-task network correspondence and novel findings on brain network activation during task fMRI in the Human Connectome Project study. Sci. Rep..

[10] Fransson P (2005). Spontaneous low-frequency BOLD signal fluctuations: An fMRI investigation of the resting-state default mode of brain function hypothesis. Hum. Brain Mapp..

[11] Fox MD (2005). The human brain is intrinsically organized into dynamic, anticorrelated functional networks. Proc. Natl. Acad. Sci. U. S. A..

[12] McAvoy M (2008). Resting states affect spontaneous BOLD oscillations in sensory and paralimbic cortex. J. Neurophysiol..

[13] Bianciardi M (2009). Modulation of spontaneous fMRI activity in human visual cortex by behavioral state. Neuroimage.

[14] Zou Q (2009). Functional connectivity between the thalamus and visual cortex under eyes closed and eyes open conditions: A resting-state fMRI study. Hum. Brain Mapp..

[15] Yan C (2009). Spontaneous brain activity in the default mode network is sensitive to different resting-state conditions with limited cognitive load. PLoS ONE.

[16] Wu L, Eichele T, Calhoun VD (2010). Reactivity of hemodynamic responses and functional connectivity to different states of alpha synchrony: A concurrent EEG-fMRI study. Neuroimage.

[17] Jao T (2013). Volitional eyes opening perturbs brain dynamics and functional connectivity regardless of visual input. Neuroimage.

[18] Liu D, Dong Z, Zuo X, Wang J, Zang Y (2013). Eyes-open/eyes-closed dataset sharing for reproducibility evaluation of resting state fMRI data analysis methods. Neuroinformatics.

[19] Patriat R (2013). The effect of resting state condition on resting-state reliability and consistency: A comparison between resting with eyes open, closed, and fixated. Neuroimage.

[20] Riedl V (2014). Local activity determines functional connectivity in the resting human brain: A simultaneous FDG-PET/fMRI study. J. Neurosci..

[21] Xu P (2014). Different topological organization of human brain functional networks with eyes open versus eyes closed. Neuroimage.

[22] Riedl V (2016). Metabolic connectivity mapping reveals effective connectivity in the resting human brain. Proc. Natl. Acad. Sci. U. S. A..

[23] Allen EA, Damaraju E, Eichele T, Wu L, Calhoun VD (2018). EEG signatures of dynamic functional network connectivity states. Brain Topogr..

[24] Wei J (2018). Eyes-open and eyes-closed resting states with opposite brain activity in sensorimotor and occipital regions: Multidimensional evidences from machine learning perspective. Front. Hum. Neurosci..

[25] Agcaoglu O, Wilson T, Wang Y-P, Stephen J, Calhoun VD (2019). Resting state connectivity differences in eyes open versus eyes closed conditions. Hum. Brain Mapp..

[26] Costumero V, Bueicheckú E, Adrián-Ventura J, Ávila C (2020). Opening or closing the eyes at rest modulates the functional connectivity of V1 with default and salience networks. Sci. Rep..

[27] Yang H (2007). Amplitude of low-frequency fluctuation within visual areas revealed by resting-state functional MRI. Neuroimage.

[28] Liang B (2014). Brain spontaneous fluctuations in sensorimotor regions were directly related to eyes open and eyes closed: Evidences from a machine learning approach. Front. Hum. Neurosci..

[29] Yuan B-K, Wang J, Zang Y-F, Liu D-Q (2014). Amplitude differences in high-frequency fMRI signals between eyes open and eyes closed resting states. Front. Hum. Neurosci..

[30] Zou Q (2015). Detecting static and dynamic differences between eyes-closed and eyes-open resting states using ALS and BOLD fMRI. PLoS ONE.

[31] Zhou Z, Wang J-B, Zang Y-F, Pan G (2018). PAIR comparison between two within-group conditions of resting-state fMRI improves classification accuracy. Front. Neurosci..

[32] Marx E (2003). Eye closure in darkness animates sensory systems. Neuroimage.

[33] Wiesmann M (2006). Eye closure in darkness animates olfactory and gustatory cortical areas. Neuroimage.

[34] Hüfner K (2008). Differences in saccade-evoked brain activation patterns with eyes open or eyes closed in complete darkness. Exp. Brain Res..

[35] Adrian ED, Matthews BHC (1934). The Berger rhythm: Potential changes from the occipital lobes in man. Brain.

[36] de Graaf T, Duecker F, Stankevich Y, ten Oever S, Sack AT (2017). Seeing in the dark: Phosphene thresholds with eyes open versus eyes closed in the absence of visual inputs. Brain Stimul..

[37] Feng Y-X (2021). The acts of opening and closing the eyes are of importance for congenital blindness: Evidence from resting-state fMRI. Neuroimage.

[38] Jeavons PM (1964). The electro-encephalogram in blind children. Br. J. Ophthalmol..

[39] Noebels JL, Roth WT, Kopell BS (1978). Cortical slow potentials and the occipital EEG in congenital blindness. J. Neurol. Sci..

[40] Hüfner K (2009). Differential effects of eyes open or closed in darkness on brain activation patterns in blind subjects. Neurosci. Lett..

[41] Liu Y (2007). Whole brain functional connectivity in the early blind. Brain.

[42] Yu C (2008). Altered functional connectivity of primary visual cortex in early blindness. Hum. Brain Mapp..

[43] Bedny M, Konkle T, Pelphrey K, Saxe R, Pascual-Leone A (2010). Sensitive period for a multimodal response in human visual motion area MT/MST. Curr. Biol..

[44] Bedny M, Pascual-Leone A, Dodell-Feder D, Fedorenko E, Saxe R (2011). Language processing in the occipital cortex of congenitally blind adults. Proc. Natl. Acad. Sci. U. S. A..

[45] Butt OH, Benson NC, Datta R, Aguirre GK (2013). The fine-scale functional correlation of striate cortex in sighted and blind people. J. Neurosci..

[46] Qin W, Liu Y, Jiang T, Yu C (2013). The development of visual areas depends differently on visual experience. PLoS ONE.

[47] Burton H, Snyder AZ, Raichle ME (2014). Resting state functional connectivity in early blind humans. Front. Syst. Neurosci..

[48] Wang D (2014). Altered resting-state connectivity in congenital blind. Hum. Brain Mapp..

[49] Murphy MC (2016). Top-down influence on the visual cortex of the blind during sensory substitution. Neuroimage.

[50] Bauer CM (2017). Multimodal MR-imaging reveals large-scale structural and functional connectivity changes in profound early blindness. PLoS ONE.

[51] Abboud S, Cohen L (2019). Distinctive interaction between cognitive networks and the visual cortex in early blind individuals. Cereb. Cortex.

[52] Loiotile, R. E. & Bedny, M. (2018). “Visual” cortices of congenitally blind adults respond to executive demands. 10.1101/390450v1 (2018).

[53] Watkins KE (2012). Language networks in anophthalmia: Maintained hierarchy of processing in ‘visual’ cortex. Brain.

[54] Butt OH, Benson NC, Datta R, Aguirre GK (2015). Hierarchical and homotopic correlations of spontaneous neural activity within the visual cortex of the sighted and blind. Front. Hum. Neurosci..

[55] Hou F, Zhou Z, Zhou J, Li H (2017). Reduction of interhemispheric functional brain connectivity in early blindness: A resting-state fMRI study. BioMed Res. Int..

[56] Pelland M (2017). State-dependent modulation of functional connectivity in early blind individuals. Neuroimage.

[57] Veraart C (1990). Glucose utilization in human visual cortex is abnormally elevated in blindness of early onset but decreased in blindness of late onset. Brain Res..

[58] Tomaiuolo F (2014). Morphometric changes of the corpus callosum in congenital blindness. PLoS ONE.

[59] Shi J (2015). Impact of early and late visual deprivation on the structure of the corpus callosum: A study combining thickness profile with surface tensor-based morphometry. Neuroinformatics.

[60] Yu C (2007). Plasticity of the corticospinal tract in early blindness revealed by quantitative analysis of fractional anisotropy based on diffusion tensor tractography. Neuroimage.

[61] Park H-J (2007). Reorganization of neural circuits in the blind on diffusion direction analysis. NeuroReport.

[62] Reislev NL, Dyrby TB, Siebner HR, Kupers R, Ptito M (2016). Simultaneous assessment of white matter changes in microstructure and connectedness in the blind brain. Neural Plast..

[63] Power JD, Schlaggar BL, Petersen SE (2015). Recent progress and outstanding issues in motion correction in resting state fMRI. Neuroimage.

[64] Power JD, Barnes KA, Snyder AZ, Schlaggar BL, Petersen SE (2012). Spurious but systematic correlations in functional connectivity MRI networks arise from subject motion. Neuroimage.

[65] Leys C, Ley C, Klein O, Bernard P, Licata L (2013). Detecting outliers: Do not use standard deviation around the mean, use absolute deviation around the median. J. Exp. Soc. Psychol..

[66] Leys C, Delacre M, Mora YL, Lakens D, Ley C (2019). How to classify, detect, and manage univariate and multivariate outliers, with emphasis on pre-registration. Int. Rev. Soc. Psychol..

[67] Benjamini Y, Hochberg Y (1995). Controlling the false discovery rate: A practical and powerful approach to multiple testing. J. R. Stat. Soc. Ser. B Stat. Methodol..

