# Supplementary material for: The effect of congenital blindness on resting-state functional connectivity revisited
Source: Sci Rep. 2021 Jun 14;11:12433. doi: 10.1038/s41598-021-91976-9 (PMC8203782; doi:10.1038/s41598-021-91976-9)
Supplement: Supplementary file 1 — Supplementary Information. [file 41598_2021_91976_MOESM1_ESM.docx]

The effect of congenital blindness on resting-state functional connectivity revisited

Maria J. S. Guerreiro^1,2,*^, Madita Linke^1^, Sunitha Lingareddy^3^, Ramesh Kekunnaya^4^, and Brigitte Röder^1^

^1^Biological Psychology and Neuropsychology, Institute for Psychology, University of Hamburg, Von-Melle-Park 11, D-20146 Hamburg, Germany.
^2^Biological Psychology, Department of Psychology, Carl von Ossietzky University of Oldenburg, D-26111 Oldenburg, Germany.
^3^Department of Radiology, Lucid Medical Diagnostics, Banjara Hills, Hyderabad 500082, Telengana, India.
^4^Child Sight Institute, Jasti V. Ramanamma Children’s Eye Care Center, Department of Pediatric Ophthalmology, Strabismus, and Neuro-Ophthalmology, L. V. Prasad Eye Institute, Kallam Anji Reddy Campus, Hyderabad 500034, Telengana, India.
^*^maria.guerreiro@uni-oldenburg.de

### Supplementary Material

In order to probe the robustness of our results – or, in other words, their independence from a particular seed definition approach –, we performed an additional analysis using an alternative parcellation scheme based on the Power 264-region atlas^1^. To recall, because we were interested in examining the effect of resting state condition on functional connectivity between sensory brain regions (rather than on whole-brain functional connectivity), we again defined seed regions in terms of primary resting-state networks (or communities). To do so, we combined all individual regions belonging to the visual system into a single *visual region,* all individual regions belonging to the auditory system into a single *auditory region*, and all individual regions belonging to the somatosensory-motor (hand) system an into a single *somatosensory region*, separately by hemisphere (see **Supplementary Fig. S1**). Data analysis was performed in the same manner as described for Study 1.

Prior to correction for multiple comparisons, paired-sample *t*-tests revealed a significant effect of resting state condition in most instances of resting-state functional connectivity (RSFC) examined, particularly between visual and somatosensory systems [FC01: *t*(27) = -2.32, *p* = .034; FC02: *t*(27) = -2.06, *p* = .049; FC03: *t*(27) = -2.99, *p* = .006; FC04: *t*(27) = -1.92, *p* = .065], but also between visual and auditory systems [FC05: *t*(27) = -1.49, *p* = .148; FC06: *t*(28) = -1.41, *p* = .170; FC07: *t*(27) = -2.56, *p* = .017; FC08: *t*(27) = -3.22, *p* = .003], whereas functional connectivity between auditory and somatosensory systems [FC09: *t*(27) = -0.14, *p* = .889; FC10: *t*(27) = -0.30, *p* = .771; FC11: *t*(27) = -1.06, *p* = .297; FC12: *t*(26) = -1.14, *p* = 2.66] – as well as within sensory systems [FC13: *t*(27) = 2.01, *p* = .054; FC14: *t*(26) = 0.62, *p* = .539; FC15: *t*(27) = -0.62, *p* = .542] – did not significantly differ across resting state conditions. As in Study 1, most of these effects were driven by a nonsignificant RSFC between visual and non-visual sensory systems during rest with eyes open [FC01: *t*(27) = 0.78, *p* = .442; FC02: *t*(27) = 0.50, *p* = .618; FC03: *t*(27) = -0.30, *p* = .764; FC04: *t*(27) = 0.51, *p* = .615; FC05: *t*(27) = -1.99, *p* = .057; FC06: *t*(27) = -2.36, *p* = .026; FC07: *t*(27) = -2.25, *p* = .033; FC08: *t*(27) = -1.33, *p* = .196], which generally tended to become significantly positive during rest with eyes closed [FC01: *t*(27) = 3.04, *p* = .005; FC02: *t*(27) = 2.91, *p* = .007; FC03: *t*(27) = 3.25, *p* = .003; FC04: *t*(27) = 3.40, *p* = .002; FC05: *t*(27) = 0.03, *p* = .997; FC06: *t*(27) = -0.31, *p* = .759; FC07: *t*(27) = 1.28, *p* = .210; FC08: *t*(27) = 2.32, *p* = .028].

After correcting for multiple comparisons, however, the effect of resting state condition survived only in the two instances of RSFC: between the right visual system and the right auditory system [FC08: *t*(27) = 3.22, *p* = .045], and between the right visual system and the left somatosensory system [FC03: *t*(27) = 2.99, *p* = .045] (**Supplementary Fig. S2**).

The present pattern of results – although overall weaker – closely replicates the results reported in the main manuscript for Study 1, providing evidence for their robustness across seed definition approaches. We argue that the effect of resting state condition is more reliable with the parcellation scheme used in the main manuscript because it restricts seed regions to relevant gray matter regions, where resting-state networks are typically contained^2^. In contrast, the parcellation scheme used here relies on 5-mm-radius spheres centered on peak coordinates derived from previous studies, which may not always provide a good spatial overlap with relevant gray matter regions across individuals.

1. Power, J. D., *et al.* Functional network organization of the human brain. *Neuron* ***72*,** 665-678 (2011).
2. Beckmann, C. F., DeLuca, M., Devlin, J. T. & Smith, S. M. Investigations into resting-state connectivity using independent component analysis. *Phil. Trans. R. Soc. B* **360,** 1001-1013 (2005).

**
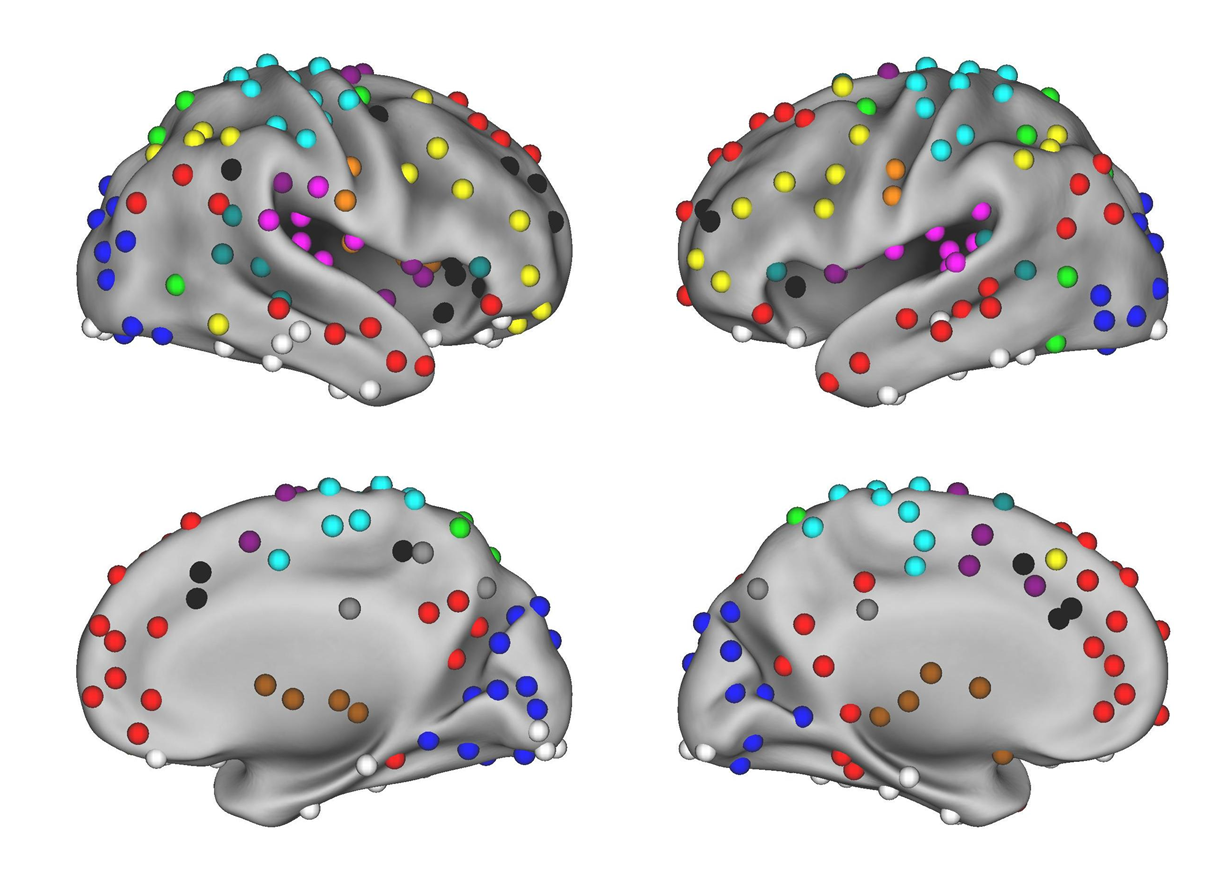
**

**Supplementary Figure S1.** Combined visual, sensory-motor, and auditory seeds were created separately by hemisphere by combining all individual visual spheres (in blue), all individual sensory-motor seeds (in cyan), and all auditory seeds (in pink) in the corresponding hemisphere. Image modified from Power *et al.*^1^, and generated using Microsoft*^®^* *PowerPoint*^TM^.

**
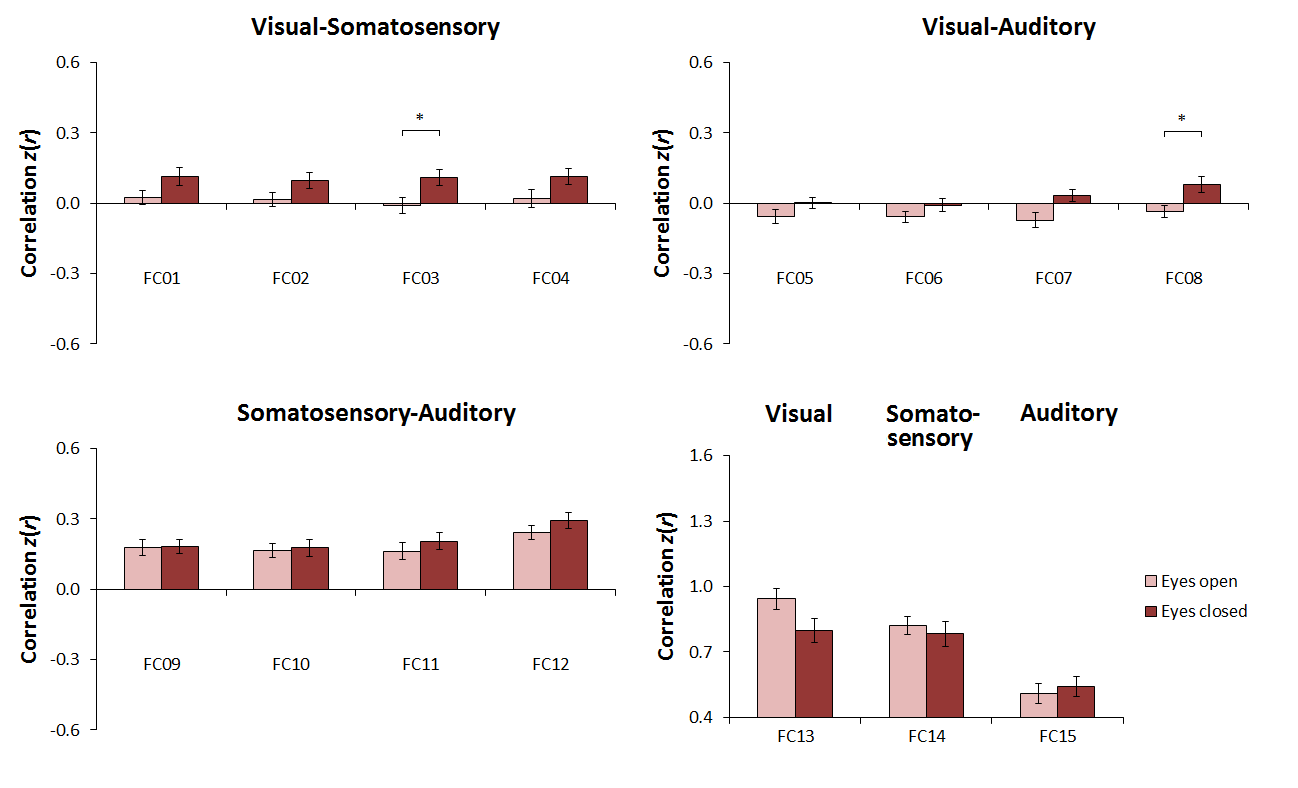
**

**Supplementary Figure S2.** Average functional connectivity and standard errors in the eyes open condition (light red bars) and in the eyes closed condition (dark red bars) for each instance of functional connectivity examined. This figure depicts the effects of resting state condition in sighted individuals, using a different parcellation scheme than that used in Study 1 (for details, see above).

**
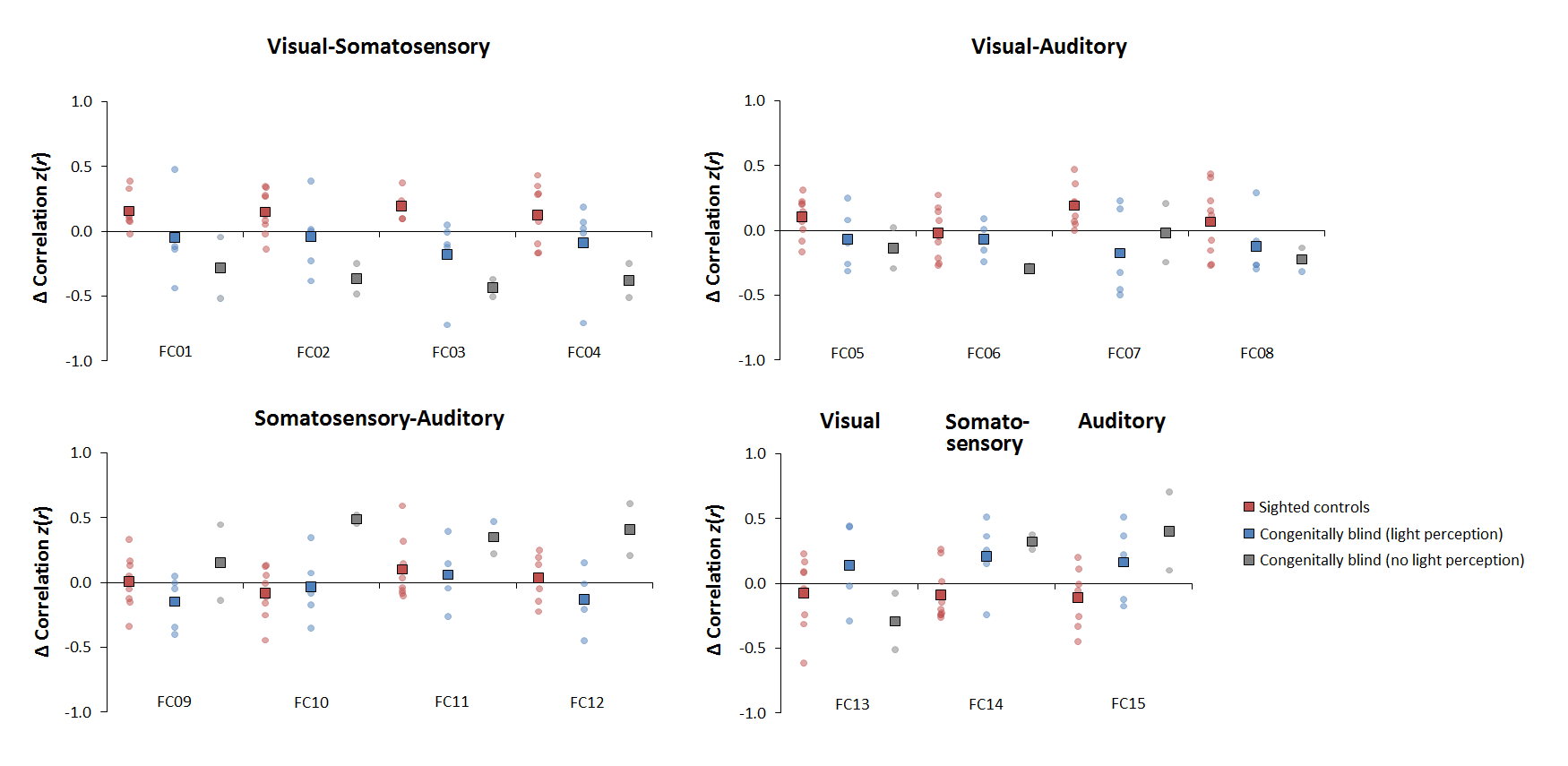
**

**Supplementary Figure S3.** Scatterplots of the difference in functional connectivity between resting state conditions (i.e., eyes closed minus eyes open) for each instance of functional connectivity examined (for details, see Table 3), plotted separately for sighted individuals (red circles), congenitally blind individuals with light perception (blue circles), and congenitally blind individuals without light perception (gray circles). The average difference in functional connectivity between resting state conditions for each group (colored squares) is depicted too for illustrative purposes.

**
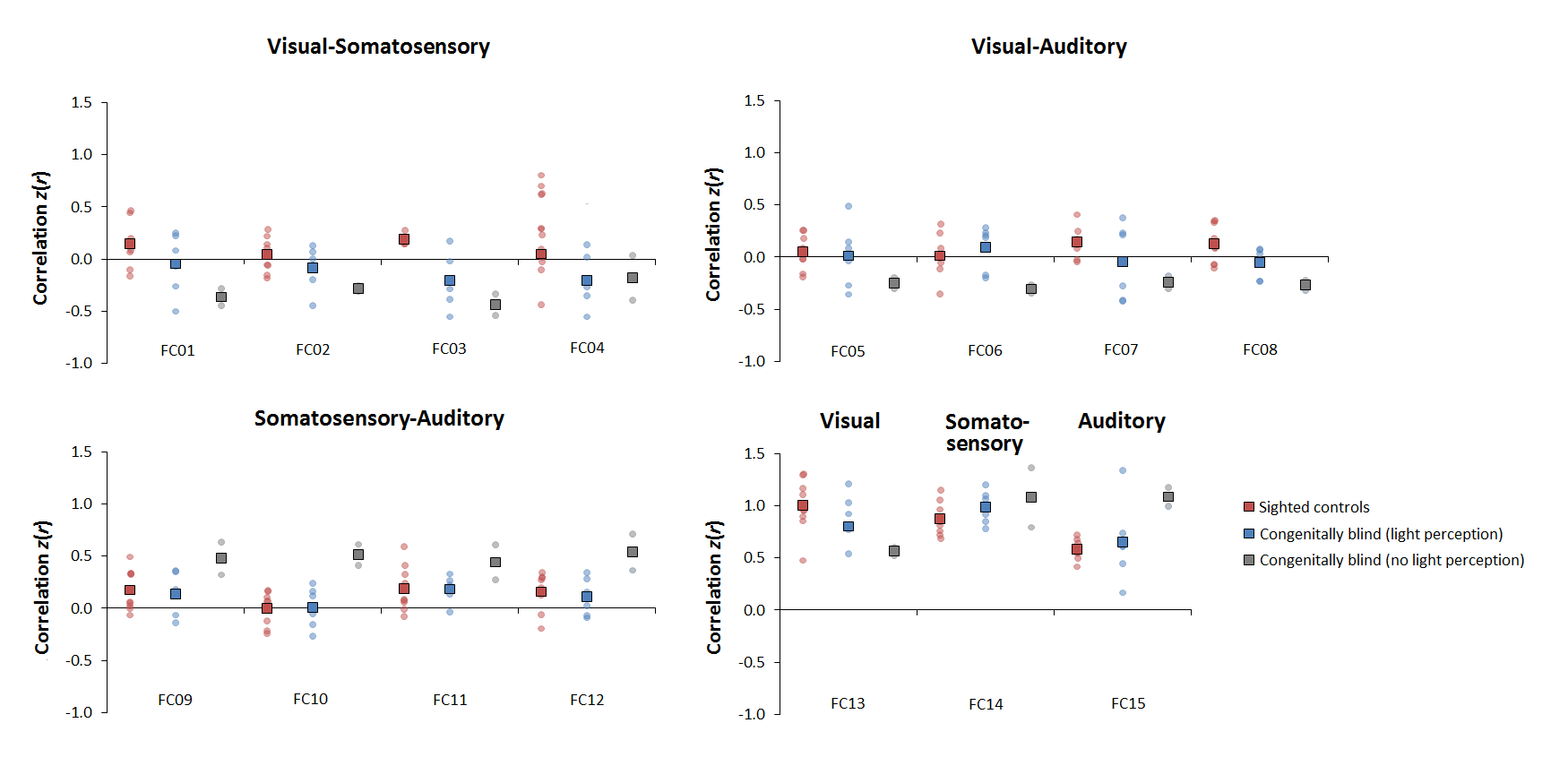
**

**Supplementary Figure S4.** Scatterplots of functional connectivity during rest with eyes closed for each instance of functional connectivity examined (for details, see Table 4), plotted separately for sighted individuals (red circles), congenitally blind individuals with light perception (blue circles), and congenitally blind individuals without light perception (gray circles). The average functional connectivity for each group during rest with eyes closed (colored squares) is depicted too for illustrative purposes.

**Supplementary Table S1.** Summary of post-hoc, one-sample *t*-tests in Study 1.

| RSFC instance | Region 1 | Region 2 | *n* ^a^ | Eyes open ^b^ | Eyes closed ^b^ |
| --- | --- | --- | --- | --- | --- |
| FC01 | VIS_LH | SOM_LH | 28 | -1.03 | 2.53 * |
| FC02 | VIS_LH | SOM_RH | 28 | -1.32 | 2.79 * |
| FC03 | VIS_RH | SOM_LH | 28 | -2.16* | 2.20 * |
| FC04 | VIS_RH | SOM_RH | 27 | -1.49 | 2.01 |
| FC05 | VIS_LH | AUD_LH | 28 | -0.89 | 1.64 |
| FC06 | VIS_LH | AUD_RH | 28 | -0.08 | 1.86 |
| FC07 | VIS_RH | AUD_LH | 23 | -2.00 | 2.98 * |
| FC08 | VIS_RH | AUD_RH | 28 | 0.47 | 3.23 * |
| FC09 | SOM_LH | AUD_LH | 27 | 4.36 * | 3.79 * |
| FC10 | SOM_LH | AUD_RH | 28 | 3.49 * | 0.98 |
| FC11 | SOM_RH | AUD_LH | 28 | 1.67 | 3.28 * |
| FC12 | SOM_RH | AUD_RH | 28 | 5.10 * | 4.64 * |
| FC13 | VIS_LH | VIS_RH | 25 | 38.90 * | 25.02 * |
| FC14 | SOM_LH | SOM_RH | 28 | 20.50 * | 23.61 * |
| FC15 | AUD_LH | AUD_RH | 28 | 18.92 * | 13.89 * |

*Note.* RSFC = resting-state functional connectivity; FC = functional connectivity; VIS = visual region; LH = left hemisphere; SOM = somatosensory region; RH = right hemisphere; AUD = auditory region.

^a^ Sample size after outlier removal. ^b^ Positive *t* values indicate that the mean was higher than zero, whereas negative *t* values indicate that the mean was lower than zero. * *p* < .050.

**Supplementary Table S2.** Summary of post-hoc, one-sample *t*-tests in Study 2.

|  |  |  |  | Sighted controls | | |  | Congenitally blind | | |
| --- | --- | --- | --- | --- | --- | --- | --- | --- | --- | --- |
| RSFC instance |  |  |  | *n* ^a^ | Eyes open | Eyes closed |  | *n* ^a^ | Eyes open | Eyes closed |
| FC01 | VIS_LH | SOM_LH |  | 9 | -0.22 | 2.05 |  | 9 | -0.05 | -1.52 |
| FC02 | VIS_LH | SOM_RH |  | 9 | -2.40 * | 0.90 |  | 9 | -0.13 | -2.21 |
| FC03 | VIS_RH | SOM_LH |  | 6 | -0.03 | 9.36 * |  | 9 | -0.93 | -3.44 * |
| FC04 | VIS_RH | SOM_RH |  | 9 | -1.67 | 0.74 |  | 9 | -0.59 | -2.57 * |
| FC05 | VIS_LH | AUD_LH |  | 9 | -1.16 | 0.92 |  | 9 | 0.69 | -0.74 |
| FC06 | VIS_LH | AUD_RH |  | 9 | 0.55 | 0.19 |  | 9 | 1.87 | -0.26 |
| FC07 | VIS_RH | AUD_LH |  | 9 | -1.84 | 3.12 * |  | 9 | 0.23 | -1.06 |
| FC08 | VIS_RH | AUD_RH |  | 9 | 0.98 | 2.05 |  | 8 | 0.22 | -2.12 |
| FC09 | SOM_LH | AUD_LH |  | 9 | 3.09 * | 2.63 |  | 9 | 4.38 * | 2.99 * |
| FC10 | SOM_LH | AUD_RH |  | 8 | 2.66 * | 0.03 |  | 9 | 1.19 | 1.69 |
| FC11 | SOM_RH | AUD_LH |  | 9 | 2.34 * | 2.60 |  | 8 | 2.21 | 4.12 * |
| FC12 | SOM_RH | AUD_RH |  | 9 | 2.97 * | 2.65 * |  | 9 | 2.32 * | 2.74 |
| FC13 | VIS_LH | VIS_RH |  | 9 | 26.27 * | 11.65 * |  | 9 | 10.95 * | 10.52 * |
| FC14 | SOM_LH | SOM_RH |  | 9 | 12.11 * | 16.85 * |  | 9 | 9.75 * | 15.48 * |
| FC15 | AUD_LH | AUD_RH |  | 7 | 7.03 * | 14.44 * |  | 9 | 5.22 * | 6.11 * |

*Note.* RSFC = resting-state functional connectivity; FC = functional connectivity; VIS = visual region; LH = left hemisphere; SOM = somatosensory region; RH = right hemisphere; AUD = auditory region.

^a^ Sample size after outlier removal. ^b^ Positive *t* values indicate that the mean was higher than zero, whereas negative *t* values indicate that the mean was lower than zero. * *p* < .050.
